# Replacing Fishmeal and Fish Oil with Complex Protein and Canola Oil: Effect on Organoleptic and Nutritional Quality of Triploid Rainbow Trout (*Oncorhynchus mykiss*)

**DOI:** 10.3390/foods13111591

**Published:** 2024-05-21

**Authors:** Yongna Song, Guoliang Sun, Fulei Wei, Zezhong Wu, Haining Tian, Yuqiong Meng, Rui Ma

**Affiliations:** 1 College of Agriculture and Animal Husbandry, Qinghai University, Xining 810016, China; 15848429897@163.com; 2 Cold-Water Fish Research Center, State Key Laboratory of Plateau Ecology and Agriculture, Qinghai University, Xining 810016, China; spy5920@126.com (G.S.); 2023990018@qhu.edu.cn (Z.W.); 3 Key Laboratory of Plateau Cold-Water Fish Culture and Eco-Environmental Conservation (Co-Construction by Ministry and Province), Ministry of Agriculture and Rural Affairs, Xining 810016, China; wfl699@163.com (F.W.); qhtianhaining@sina.com (H.T.); 4 College of Eco-Environmental Engineering, Qinghai University, Xining 810016, China

**Keywords:** triploid rainbow trout, substitution, quality, growth, fish meal, fish oil

## Abstract

A twelve-week feeding experiment was undertaken to explore the impact of substituting dietary fish meal (FM) and fish oil (FO) with complex protein (CP) and canola oil (CO) in the diet of triploid rainbow trout on the quality of their fillets. The control diet (F100) contained FM (60%) and FO (18.6%) as the main protein and lipid sources. Based on this, 50% and 100% of FM and FO were substituted by CP and CO and they were named as F50 and F0, respectively. The results showed that there were no significant differences in the specific growth rates, condition factors, gutted yields, fillet yields and yellowness values as the substitution levels increased (*p* > 0.05). The F50 treatment obtained the highest values of fillet springiness and chewiness, improved the umami and bitter taste of the fillets by increasing the contents of inosine-5′-monophosphate and histidine, and increased lipid, protein, C18: 1n-9 and C18: 2n-6 contents (*p* < 0.05). The F0 treatment obtained the highest values of fillet hardness and pH, attenuated the sweet taste of the fillets by decreasing the content of glycine, and decreased the contents of EPA and DHA (*p* < 0.05). Both F50 and F0 treatments could increase the redness value, decrease the lightness and hue values of fillets, and increase the odor intensity, resulting in the typical fillet odors of green, fatty, orange and fishy (*p* < 0.05). In general, 50% and 100% of FM and FO substitution did not affect the growth of trout, but it did affect quality. Compared to the F100 treatment, the fillet quality of the F0 treatment was similar to the F50 treatment and could improve the appearance and odor intensity of the fillets. However, the difference was that the F50 treatment increased the springiness, umami, bitterness and lipid nutritional value of the fillets, but the F0 treatment increased the hardness, decreased the sweetness, and decreased the lipid, EPA and DHA contents of the fillets.

## 1. Introduction

As global aquaculture has grown rapidly in recent decades, so has the demand for aquatic feed [1]. The traditional aquatic feed utilizes fish meal (FM) and fish oil (FO) as the main protein and lipid sources, which provide essential nutrients for the growth of farmed fish. Due to limited marine sources, identifying alternatives to FM and FO is particularly important for the sustainable growth of aquaculture [2]. Terrestrial sources are the preferred alternatives for the aquatic feed industry. Recent decades have seen increasing utilization of terrestrial sources in aquatic feed, and numerous growth trials have been conducted to evaluate the applicability of terrestrial sources for different varieties of aquatic species; however, results have varied [3,4]. Rainbow trout (*Oncorhynchus mykiss*) ranks among the most extensively cultivated cold-water fish species globally, and is renowned for the high nutritional quality of its fillets [5]. As a carnivorous fish, rainbow trout seem to be well adapted to terrestrial food sources. Studies have shown that partial substitution of FM with a terrestrial protein source has no significant impact on the growth of rainbow trout [6,7]. Our previous studies have shown that FO substituted completely by canola oil (CO) did not impact the growth of triploid rainbow trout, but did alter fish quality [8]. Therefore, it is important to evaluate feed ingredients based not only on growth performance but also fish quality.

Fish quality has now become an important factor affecting consumers’ purchase desire and the economic value of fish, and it can be described by a series of quality-related parameters. Fish weight, shape and color are the main indicators used to evaluate the appearance quality of fish. Hardness, springiness, chewiness and so on are the main indicators of the texture of fish fillets. Fish flavor, including fillet odor and taste, can be described by the composition of flavor substances, while the nutritional quality of fish is mainly indicated by the protein, fat and fatty acid content of fillets. The understanding of the effects of aquatic feed ingredients on fish quality is still very limited. Previous studies on rainbow trout showed that FO and FM substitution could reduce the highly unsaturated n-3 fatty acid compositions of fish fillets [9,10]; however, systematic and comprehensive evaluation of FM and FO substitution in feed on the quality of triploid rainbow trout is still lacking.

Therefore, the objective of this study was to use analytical techniques to assess the impacts of the substitution of dietary FM and FO on the sensory quality and nutritional quality of triploid rainbow trout fillets.

## 2. Materials and Methods

### 2.1. Animals, Experimental Diets and Feeding Trial

This study adhered strictly to the standard operating procedures outlined in Qinghai University’s guide for the use of experimental animals, and the research protocol was thoroughly reviewed and approved by Qinghai University’s ethical committee. All female triploid rainbow trout utilized in the study were sourced from local fisheries in Qinghai, China.

According to our previous studies, conducted by Ma et al. [11] and Meng et al. [12], on the protein and lipid requirements of triploid rainbow trout, we formulated three diets, as shown in Table A1. For the control treatment (F100), FM and FO served as the primary sources of protein and lipids. Based on this, both were substituted with complex protein (CP, including soybean meal, soy protein concentrate, corn gluten meal, wheat gluten and blood cell powder) and CO in the proportion of 50% and 100%, respectively, and they were named as F50 and F0, respectively. Tongwei Co., Ltd., Chengdu, China manufactured all experimental diets in the form of 4 mm expanded feed pellets. The methods for their preparation and storage followed previously established protocols [11].

The experimental juveniles were all obtained from the same batch of commercially hatched triploid rainbow trout, with an initial body weight of 208 g. After starvation, they were randomly assigned to 12 freshwater net cages (3 m length × 3 m width × 6 m depth) with 100 fish in each. Each feed fed four random net cages as replicates. During the 12 weeks of feeding, experimental diets were fed at 08:30 and 16:30 until apparent satiety. The water temperature fluctuated between 9.0 °C to 17.0 °C while the dissolved oxygen content exceeded 7 mg/L. After feeding for twelve weeks, all fish were starved for 2 days before slaughter. The final weights of F100, F50 and F0 were 769 g, 764 g and 787 g, respectively. Three fish per cage were randomly selected and euthanized under excessive anesthesia. Then, the left gill of each fish was cut and bled. After their body weights and lengths were measured, they were gutted, filleted and weighed. The left fillet samples were carried back to the laboratory in zip-lock bags in a covered polystyrene ice box. Subsequently, the fillets were analyzed for texture, pH, water holding capacity (WHC) and fillet color. Then, the fillets were cut into specific pieces according to the requirements and put into a zip-lock bag and stored in the refrigerator at −80 °C for the subsequent determination.

### 2.2. Biometric Parameters Calculation

Based on the sampled data, the condition factors, gutted yields and fillet yields were calculated by referring to Meng et al. [12]. The specific growth rate was calculated with reference to Meng et al. [13]. The calculation formulae are as follows:

Condition factor (%) = [body weight (g)/body length (cm)]^3^ × 100 (1)

Gutted yield (%) = [carcass gutted weight (g)/body weight (g)] × 100(2)

Fillet yield (%) = [fillets weight (g)/body weight (g)] × 100(3)

Specific growth rate (SGR, %/day) = 100 × ln [final body weight (g)/initial body weight (g)]/days of the experiment(4)

### 2.3. Fillet Color, Texture, PH and Water Holding Capacity Measurements

We selected two points on the back and tail of the fillet for color measurement (CR-400, Konica Minolta, Tokyo, Japan), including lightness (L*), redness (a*) and yellowness (b*). After measurement, the chroma (C*_ab_) and hue (*H*^°^_ab_) values were calculated with reference to Meng et al. [13]. The calculation methods are as follows:

C*ab = [a*^2^ + b*^2^]^1/2^ (5)

*H^°^ab* = arctan [b*/a*] (6)

The texture-related indicators were measured using a physical property analyzer (TMS-PRO, FTC, Sterling, VA, USA) at the abdomen and back of each fish fillet, and the instrument squeezed each point twice to measure the muscle hardness, adhesiveness, springiness, cohesiveness, chewiness and so on. The pH value each fillet was determined using a pH meter (S220, Mettler Toledo, Zurich, Switzerland) at the same position after extrusion of the food physical property analyzer. Determination of the liquid losses and water holding capacities of the fish was conducted with reference to Meng et al. [12]. The calculation methods are as follows:

Liquid losses = (m_1_ − m_0_)/m × 100% (7)

Water holding capacity = 100% − Liquid losses(8)

In the above formulae, m is the weight of the fish before extrusion, m_0_ is the weight of the filter paper, and m_1_ is the weight of the filter paper after extrusion.

### 2.4. Biochemical Compositions Analysis

The protein and lipid contents of the diets were detected by the AOAC (1995) standard method [14]. The fillets’ contents of protein, ash, lipids and moisture were measured by the Kjeldahl method (Dumas Nitrogen Analyzer, Dumatec^TM^ 8000, FOSS, Hilleroed, Denmark), the combustion method at 550 °C (HXM2000/D, HuaXing, Hunan, China), the chloroform-methanol extraction method and the freeze-drying method, respectively. Refer to Ma et al. [15] for the determination methods for water, salt-soluble protein content and collagen contents of fish fillets. The muscle glycogen contents and lactic dehydrogenase (LDH) activities were measured by Kit (Nanjing Jiancheng, Nanjing, China).

### 2.5. Volatile Compounds Analysis

The instrument used for the determination of volatile odorants was a gas chromatography–mass spectrometer (GC-MS, QP2020, Shimadzu, Kyoto, Japan) equipped with an automatic solid-phase microextraction (SPME) system (AOC-6000, CTC, Zwingen, Switzerland). Refer to Ma et al. [16] for the determination and calculation methods. The calculation methods are as follows:

OAV = C_i_/OT_i_ (9)

In the formula, the C_i_ and OT_i_ were represented by the relative concentration of volatile odorants (ng/g) and the threshold of volatile odorants (ng/g), respectively.

### 2.6. Non-Volatile Compounds Analysis

The extraction and detection of nucleotides were conducted with reference to Ma et al. [15].

Free amino acid measurements were conducted with reference to Chen and Zhang [17]. 1 g of muscle lyophilized powder was fully mixed with 4 mL of 10% sulfonyl salicylic acid solution, stood for 5 min at 4 °C and centrifuged, then separated by a high-performance liquid chromatograph (HPLC; 1260 Infinity II, Agilent, Santa Clara, CA, USA).

The equivalent umami concentrations (EUC) were calculated based on the description of Liu et al. [18], and the formula is as follows:

EUC (gMSG/100 g) = Σa_i_b_i_ + 1218 (Σa_i_b_i_) (Σa_j_b_j_) (10)

In the formula, a_i_ is the concentration (g/100 g) of each umami amino acid in terms of aspartic acid and glutamic acid; b_i_ is the relative umami concentration (RUC) for each umami amino acid versus monosodium glutamate (MSG; aspartic acid, 0.077 and glutamic acid, 1); a_j_ is the concentration (g/100 g) of each umami adenosine-5′-monophosphate (AMP), guanosine-5′-monophosphate (GMP) and inosine-5′-monophosphate (IMP); b_j_ is the RUC for each umami 5′-nucleotide versus IMP (IMP, 1; GMP, 2.3 and AMP, 0.18); and 1218 is a synergistic constant.

Organic acids were extracted and measured with reference to Liu et al. [18], with some modification. We accurately weighed 1 g of muscle into a 15 mL centrifuge tube, added ultra-pure water to 5 mL and homogenized. After homogenizing, the mixtures were centrifuged at 13,000 r/min for 15 min, and HPLC analysis was conducted on the supernatant. The succinic acid, oxalic acid, lactic acid and maleic acid were measured using a standard kit (Supelco, Bellefonte, PA, USA).

The Ca^2+^, Na^+^, K^+^, and Mg^2+^ levels were determined by ICP-MS (ICAP RQ, Thermo, Waltham, MA, USA). The methods for the measurement of phosphate and chloride ion concentrations using an ion chromatograph system (ICS-1100, Dionex, Sunnyvale, CA, USA) referred to Guan et al. [8].

The contributions of various flavor compounds in fish fillets were evaluated by calculating their taste activity values (TAVs). When the TAV ≥ 1, the substance is considered to be active in food taste. The calculation method is as follows:

TAV = C_i_/TT_i_ (11)

In the formula, C_i_ and TT_i_ represented the concentrations of the compounds related to taste and the threshold values of the taste compounds reported in the literature, respectively.

### 2.7. Fatty Acids Analysis

Refer to Ma et al. [15] for the methods of methylation and subsequent analysis of fatty acids by a gas chromatograph and mass spectrogram (GC–MS, QP2020, Shimadzu, Kyoto, Japan). After separation, the absolute content of each fatty acid was obtained according to the concentration of the internal standard and the ratio of the peak area between the target fatty acid and the internal standard.

### 2.8. Statistical Analysis

The experimental data were presented in the form of mean ± standard error and were analyzed and processed using SPSS version 25.0 statistical software. To calculate significant differences (*p* < 0.05), multiple comparisons were conducted using Tukey’s test. Principal component analysis and cluster analysis were executed using SPSS version 25.0, with the results presented using the Origin 2019 software.

## 3. Results

### 3.1. Fillet Appearance Quality

As the dietary substitution levels increased (Table 1), no significant differences in specific growth rates, condition factors, gutted yields, fillet yields or b* values were observed among all the treatments (*p* > 0.05). F100 treatment obtained the highest values of L* and H^°^_ab_ but the lowest values of a* and C*_ab_ in fish fillets (*p* < 0.05).

### 3.2. Fillet Texture

In terms of physical properties (Table 2), there were no significant differences in breaking force and WHC (*p* > 0.05). The F50 treatment obtained the highest muscle thickness, cohesiveness, springiness and chewiness values significantly, and the lowest adhesiveness value (*p* < 0.05) significantly. Hardness and pH showed the highest values significantly in the F0 treatment (*p* < 0.05).

In terms of biochemical compositions (Table 2), there were no significant differences for the fillet content of moisture, ash, water soluble protein, glycogen and LDH activity (*p* > 0.05). The F100 treatment obtained the significantly lowest protein content (*p* < 0.05), while the F0 treatment obtained the significantly lowest contents of lipids and total hydroxyproline (HYP) in the fillets (*p* < 0.05). There were no differences between the F100 and F50 treatments (*p* > 0.05). Furthermore, the F0 treatment obtained the significantly lowest average values of salt soluble protein and hydroxyproline content (*p* < 0.05).

### 3.3. Fillet Odor

In this study, a total of 19 compounds related to odor-active compounds (OAV ≥ 1) were detected (Table 3). No significant differences in (E)-2-nonenal, (E,Z)-2,6-nonadienal, (Z)-4-heptenal, decanal, 2,3-octanedione, 1-heptanol, undecanal, pentanal, 3,5-octadien-2-one and (E,E)-2,4-heptadienal contents were observed in the fillets with the experimental diets (*p* > 0.05). ∑n-3 derived content was the significant lowest value observed for the F100 treatment (*p* < 0.05). The concentrations of nonanal, 1-octen-3-ol, (E)-2-decenal, heptanal, 2-ethyl-furan, (E)-2-octenal, 2-pentyl-furan, ∑n-6 and ∑n-9 derived in the F0 treatment were significantly higher than those in the F100 treatment (*p* < 0.05).

As the dietary substitution levels increased, the fillet concentrations were significantly different, with the F0 treatment obtaining a significantly higher value than the F100 treatment (*p* < 0.05). Similarly, this trend was also found for the contents of hexanal and total odor-active compounds (TOAC), which increased markedly and reached the highest value significantly in the F0 treatment (*p* < 0.05).

### 3.4. Fillet Taste

There were no significant differences in AMP, threonine, proline, lysine, methionine, phenylalanine, tyrosine, oxalic acid, lactic acid, succinic acid, Na^+^, K^+^, Mg^2+^ and PO_4_^3-^ content were observed in the fillets (*p* > 0.05, Table 4). The IMP, glutamic acid, sum of umami taste compounds (SUTC) and EUC contents showed the highest values significantly in the F50 treatment (*p* < 0.05). The highest contents of GMP and aspartic acid were observed in the F100 treatment (*p* < 0.05). The F100 and F50 treatments obtained the highest contents significantly of glycine and sum of sweet taste compounds (SWTC) (*p* < 0.05), and the F50 and F0 treatments obtained the highest alanine contents significantly (*p* < 0.05). The F0 treatment obtained the significantly lowest content of glycine, while the F100 treatment obtained the significantly lowest alanine content (*p* < 0.05). The valine, leucine and isoleucine contents in the F100 treatment showed the significantly lowest values (*p* < 0.05). The F50 treatment showed the highest histidine and sum of bitter taste compounds (SBTC) contents significantly (*p* < 0.05). The arginine content of the F0 treatment showed the highest value significantly. The highest content of maleic acid was found in the F100 treatment (*p* < 0.05). The fillet concentrations of Ca^2+^ and Cl^-^ increased gradually, with significant higher values observed in the F0 treatment than in the F100 treatment (*p* < 0.05).

### 3.5. Fillet Nutritional Values

There were no significant differences between the TI and PUFA/SFA contents of all the treatments (*p* > 0.05, Table 5). The F0 treatment obtained the lowest contents of EPA, DHA, EPA + DAH, LC-PUFA and ∑n-3 (*p* < 0.05), while no significant differences were found between the F50 and F100 treatments (*p* > 0.05).The F50 treatment significantly increased the contents of C18: 1n-9, C18: 2n-6, TFA, SFA, MUFA, PUFA and ∑n-6 compared to the other treatments (*p* < 0.05), and the F100 treatment obtained the highest contents of n-3 PUFA/n-6 PUFA and AI (*p* < 0.05).

### 3.6. Principal Component Analysis

**Figure 1:**
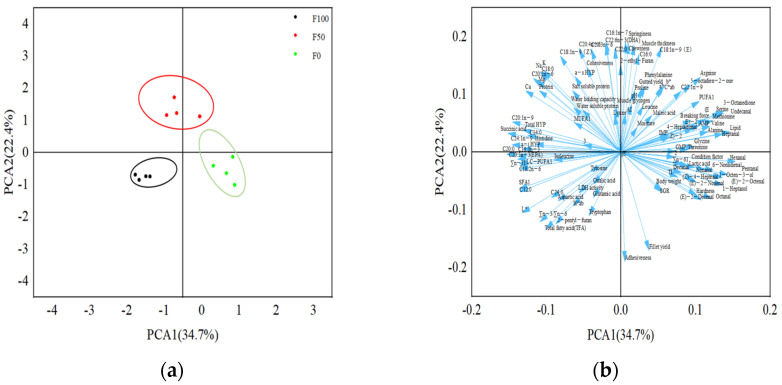
Principal component analysis (PCA) and cluster analysis of fillet quality parameters in the control (FO), 50% (F50) and 100% (F100) substitution groups. PCA and cluster analysis of the results from fish samples showing the first two principal components (PCA1 and PCA2). In the score plot (**a**), there are four pooled samples within each group, each sample was consisted of three fish (*n* = 4); In the correlation loading plot of the measured variables (**b**), there are 114 arrows and each arrow point to one quality variable (indicator).

Figure 1 shows the principal component analysis (PCA) of the 114 quality indicators of triploid rainbow trout fed with the experimental diets. From Figure 1a can be seen that the quality profiles of fillets under different substitution levels were significantly different and they can be grouped independently. Each arrow in the correlation loading plot in Figure 1b signifies a quality indicator, with arrows pointing in the same or opposite direction indicating that there is a positive or negative correlation between them. The separation of treatment F50 from the other treatments on PCA2 can be observed in Figure 1b, while the separation of treatment F100 from the other treatments on PCA1 is evident. The indicators distributed on PCA1 and PCA2 in Figure 1a are consistent with the research results.

## 4. Discussion

This study showed that feed with 50% and 100% canola oil and complex protein levels had no significant effect on the growth, condition factors, gutted yields and fillet yields of triploid rainbow trout. Previous studies have shown similar results [9,19,20].

As one of the most intuitive indicators used to evaluate appearance quality, fillet color may affect the value of fish and the choice of consumers [21]. Especially for salmonids, consumers prefer the orange color of fish fillets caused by astaxanthin deposition. The results of this study showed that CO and CP substitution in diets significantly increased the redness of the color of fish fillets, resulting in higher values of C*_ab_ in the F50 treatment and of H*_ab_ values in the F100 treatment. Previous studies have found that dietary fatty acid profiles could affect the profile of fillets and their the lipid nutritional value [22]. In this study, the F100 treatment obtained the highest content of n-3 fatty acids, which were easily oxidized, and astaxanthin was a strong antioxidant. Therefore, the F100 treatment required more astaxanthin to antioxidize, resulting in the redness of fish fillets in the F100 treatment being lower than that in the F50 and F0 treatments. Meanwhile, the PCA diagram reveals positive correlations between the a* and C*_ab_ values and the lipid contents of fish fillets, further corroborating the aforementioned hypothesis.

The texture of fillets plays an important role in evaluating the quality of fish [23]. In this research, the fillets in the F0 treatment obtained the highest value of hardness, while the fillets in the F50 treatment obtained the highest cohesiveness, springiness and chewiness values. These results indicated that the fillets of fish fed with substitutions may have firmer and more elastic texture when chewed. The relatively lower lipid content in the F0 treatment and higher protein contents in both the F50 and F0 treatments may be partly responsible. Moreover, the rapid decrease in muscle pH after death is thought to be one of the reasons for the soft texture of fish fillets [24]. Thus, the highest pH value significantly may partly explain the harder texture of fish fillets from the F0 treatment. Previous studies have shown that changes in pH are associated with the production of lactic acid, which results from the anaerobic degradation of glycogen in fish [25]. In this study, principal component analysis showed that there was a negative correlation between pH and lactic dehydrogenase in fish fillets, although both muscle glycogen and lactic dehydrogenase activity showed no significant differences. In summary, the substitution in the feed could improve fillet texture.

The production of flavor is mainly caused by odor substances, including volatile small molecular compounds. Odor formation is related to volatile compound concentration and odor threshold. When OAV ≥ 1 it contributes to the overall odor profile of the fish [16]. Our laboratory has previously conducted research on the types of odor-active compounds found in triploid rainbow trout (21 species) [26], and 19 of them were confirmed in this study. Among all the odor-active compounds, the top five that contributed to the odor of triploid rainbow trout were nonanal, octanal, (E)-2-nonenal, 1-octen-3-ol and hexanal, which was consistent with our previous study [16]. Furthermore, this study also found that nine odor-active compounds, including nonanal, octanal, 1-octen-3-ol, hexanal, (E)-2-decenal, heptanal, 2-ethyl-furan, (E)-2-octenal and 2-pentyl-furan, increased as the substitution levels increased. By searching the literature, we found that nonanal and octanal were formed by the decomposition reactions of 10- and 11-hydroperoxides after the autoxidation of 18:1n-9 [27], 1-octen-3ol was formed by 12-lipoxygenase during 20:4n-6 oxidation, and hexanal was formed by 13-hydroperoxides during 18:2n-6 oxidation [28]. Meanwhile, in the PCA diagrams for the current study, a negative correlation appeared to exist between the content of these odor compounds and polyunsaturated fatty acids in the fillets. The formation of odor compounds needs further study. Moreover, the present study also found that an ascending order of fillet TOAC was F100 < F50 < F0. This is similar to our previous research, in which the volatile compound profile of trout fillets was altered by dietary canola oil substitution [8]. Utilizing the data derived for Σn-3, Σn-6 and Σn-9, this study found that the primary production of odor-active compounds was by n-6 fatty acids, followed by n-9 fatty acids and n-3 fatty acids. Overall, the substitution with CO and CP was observed to intensify the odor of the fillet, particularly in the F0 treatment.

Taste is a sensation caused by non-volatile taste substances that can be perceived by the human taste system, and it is characterized by sourness, bitterness, saltiness, sweetness and umami. For umami taste, IMP content, taste active compounds (TAV ≥ 1) and EUC values reached the highest levels in the F50 treatment, which had no significant difference with the F0 treatment. The results indicated that CO and CP substitution could increase the fillet umami taste of triploid rainbow trout. For sweet taste, several free amino acids contributed to the sweetness of the fillets; however, no sweet taste active compound was found. SWTC content was higher in the F100 and F50 treatments compared to the F0 treatment. For bitter taste, both histidine content and SBTC were highest in the F50 treatment, indicating that 50% substitution could increase the bitterness of trout fillets. Organic acids are closely associated with the production of sour taste [29]. Increased substitution levels did not affect the sourness of the fillets, except that a slightly decreased maleic acid content was observed in this study. The change in Cl^−^ content (saline taste active compound) and SATC indicated that the saline taste of trout fillets was not affected. Overall, the F50 and F0 treatments showed the characteristics of umami and bitter taste.

Generally, the composition of fatty acids in fish fillets were easily influenced by the diets [22]. The F50 treatment contained the highest contents of C18: 1n-9 and C18: 2n-6, indicating that 50% substitution enhances the nutritional value of the fillets. Conversely, in the F0 treatment, these two fatty acids’ contents decreased, possibly due to imbalances in fatty acid composition and excessive consumption as precursor substances. Surprisingly, the F50 and F100 treatments had no significant difference in EPA and DHA content, suggesting that excessive levels of EPA and DHA may not facilitate deposition. Previous studies have shown that the proposed ratios of n-3/n-6 PUFA and PUFA/SFA in foods are 0.25 and 0.45 [30,31]. in the findings from this study basically conform to these recommended values. In addition, studies have shown that AI and TI indices have negative impacts on human health [32], and the proposed values of both are less than 1.0 and 0.5, respectively. All indicators in this study were higher than the proposed values, and the F0 treatment was higher compared to the F100 and F50 treatments. In general, CO and CP substitution positively influenced the deposition of lipids and fatty acids in fish fillets, which is beneficial to human health.

## 5. Conclusions

In general, 50% and 100% of FM and FO substitution did not influence trout growth, but it did affect quality. Compared to the F100 treatment, the fillet quality of the F0 treatment was similar to the F50 treatment, as both could improve the appearance and odor intensity of the fillets. However, the difference was that the F50 treatment increased the springiness, umami, bitterness and lipid nutritional value of the fillets, but the F0 treatment increased the hardness and decreased the sweetness, lipid content, EPA content and DHA content of the fillets.

## Figures and Tables

**Table 1:** Biometric parameters and fillet colors of triploid rainbow trout fed with the different diets.

	F100	F50	F0
Biometric parameters			
Specific growth rate (SGR) ^1^	1.56 ± 0.22	1.55 ± 0.01	1.58 ± 0.02
Condition factor (%) ^1^	1.86 ± 0.03	1.89 ± 0.03	1.96 ± 0.02
Gutted yield (%) ^1^	89.34 ± 0.41	90.04 ± 0.27	89.53 ± 0.29
Fillet yield (%) ^1^	61.90 ± 0.37	60.55 ± 0.59	62.13 ± 0.54
Fillet color			
L* ^2^	51.76 ± 0.90 ^b^	44.01 ± 1.32 ^a^	43.64 ± 0.58 ^a^
a* ^2^	11.16 ± 0.59 ^a^	12.95 ± 0.43 ^b^	12.25 ± 0.37 ^b^
b* ^2^	19.61 ± 0.83	21.41 ± 0.40	20.70 ± 0.52
C*_ab_ ^3^	20.47 ± 1.13 ^a^	24.25 ± 0.95 ^b^	22.81 ± 0.51 ^ab^
*H^°^_ab_* ^3^	62.45 ± 0.72 ^b^	60.78 ± 0.85 ^a^	59.83 ± 0.52 ^a^

Values (mean ± standard error, *n* = 4, N = 16) in the same row with different superscripts are significantly different (*p* < 0.05; Tukey’s test). ^1^ Condition factor = [body weight(g)/body length(cm)]^3^ × 100; Gutted yield = [carcass gutted weight (g)/body weight (g)] × 100; Fillet yield = [fillets weight (g)/body weight (g)] × 100; Specific growth rate (SGR, %/day) = 100 × ln [final body weight (g)/initial body weight (g)]/days of the experiment. ^2^ L*, a*, b*, C*_ab_ and H*_ab_ represent lightness, redness, yellowness, chroma and hue, respectively. ^3^ C*_ab_ = [a*^2^ + b*^2^]^1/2^; *H^°^ab* = tan^−1^[b*/a*].

**Table 2:** Fillet texture of triploid rainbow trout fed with the diets with different substitution levels.

	F100	F50	F0
Physical properties Texture profile analyses (TPA)			
Muscle thickness (mm)	10.33 ± 0.29 ^a^	15.29 ± 0.75 ^b^	11.32 ± 0.31 ^a^
Breaking force (N)	3.20 ± 0.26	3.61 ± 0.53	4.09 ± 0.22
Hardness (N)	4.19 ± 0.20 ^a^	4.00 ± 0.23 ^a^	5.33 ± 0.18 ^b^
Adhesiveness (mJ)	2.59 ± 0.14 ^b^	0.59 ± 0.06 ^a^	2.52 ± 0.10 ^b^
Cohesiveness	0.20 ± 0.01 ^a^	0.29 ± 0.03 ^b^	0.18 ± 0.01 ^a^
Springiness (mm)	1.78 ± 0.09 ^a^	7.10 ± 0.55 ^b^	2.53 ± 0.12 ^a^
Chewiness (mJ)	1.48 ± 0.13 ^a^	7.48 ± 0.65 ^b^	2.51 ± 0.19 ^a^
Water holding capacity (WHC, %) ^1^	90.39 ± 0.49	90.87 ± 0.36	89.33 ± 0.49
pH	6.32 ± 0.01 ^a^	6.30 ± 0.01 ^a^	6.47 ± 0.05 ^b^
Biochemical compositions			
Moisture (%)	69.57 ± 0.05	69.82 ± 0.33	69.68 ± 0.50
Ash (%)	1.36 ± 0.05	1.24 ± 0.05	1.33 ± 0.05
Lipid (%)	7.89 ± 0.18 ^b^	8.44 ± 0.49 ^b^	6.31 ± 0.34 ^a^
Protein (%)	19.13 ± 0.35 ^a^	20.26 ± 0.24 ^b^	20.92 ± 0.08 ^b^
Water soluble protein (mg/g muscle)	61.73 ± 2.06	66.66 ± 0.79	58.23 ± 4.34
Salt soluble protein (mg/g muscle)	79.08 ± 1.47 ^ab^	82.23 ± 1.92 ^b^	74.53 ± 1.91 ^a^
a-s HYP (mg/g muscle) ^2^	0.04 ± 0.00 ^ab^	0.05 ± 0.00 ^b^	0.04 ± 0.00 ^a^
a-i HYP (mg/g muscle) ^2^	0.13 ± 0.01 ^b^	0.11 ± 0.01 ^ab^	0.07 ± 0.01 ^a^
Total HYP (mg/g muscle) ^2^	0.18 ± 0.01 ^b^	0.16 ± 0.01 ^b^	0.11 ± 0.01 ^a^
Muscle glycogen (mg/g muscle)	0.32 ± 0.02	0.38 ± 0.04	0.33 ± 0.05
LDH activity (U/gprot) ^3^	21,089.96 ± 2994.98	15,594.32 ± 2360.29	15,126.40 ± 3063.59

Values (mean ± standard error, *n* = 4, N = 16) in the same row with different superscripts are significantly different (*p* < 0.05; Tukey’s test). ^1^ WHC (%) = 100 × [initial weight (g) − final weight (g)]/initial weight (g). ^2^ a-s HYP: alkaline-soluble hydroxyproline; a-i HYP: alkaline-insoluble hydroxyproline; total HYP: total hydroxyproline; ^3^ LDH activity: lactic dehydrogenase activity.

**Table 3:** Fillet odors of triploid rainbow trout fed the diets with different substitution levels.

Odor-Active Compounds ^1^	Odor Description ^2^	F100	F50	F0
		ng/g Muscle (OAV ^1^)		
Nonanal	Geranium, fishy, plastic, orange, green, fatty	174.49 ± 12.64 (158.63) ^a^	200.28 ± 20.79 (182.07) ^a^	429.76 ± 7.43 (390.69) ^b^
Octanal	Sweet, orange, floral, pungent, green, fatty	115.48 ± 15.46 (164.96) ^a^	118.67 ± 9.14 (169.53) ^a^	186.35 ± 15.68 (266.22) ^b^
(E)-2-Nonenal	Moss, woody, floral, green, fruity	6.61 ± 0.75 (82.58)	7.08 ± 1.17 (88.41)	8.93 ± 0.85 (111.65)
1-Octen-3-ol	Earthy, mushroom, fermented	98.76 ± 23.8 (65.84) ^a^	131.43 ± 30.03 (87.62) ^ab^	231.32 ± 34.89 (154.21) ^b^
Hexanal	Garlic, green, grassy, pungent, fatty, fishy	228.03 ± 50.11 (50.67) ^a^	520.26 ± 38.10 (115.61) ^b^	1004.63 ± 58.20 (223.25) ^c^
(E)-2-Decenal	Orange, fatty	10.10 ± 0.94 (33.68) ^a^	10.13 ± 0.48 (33.78) ^a^	17.07 ± 3.17 (56.89) ^b^
Heptanal	Green, floral, fatty, pungent, fishy, nutty, chocolate, mushroom	65.88 ± 18.58 (23.53) ^a^	159.14 ± 38.59 (56.84) ^ab^	203.10 ± 13.37 (72.53) ^b^
(E, Z)-2,6-Nonadienal	Cucumber, floral	8.88 ± 2.19 (11.1)	8.58 ± 0.28 (10.73)	8.54 ± 0.83 (10.67)
(Z)-4-Heptenal	Fishy, boiled potato	37.04 ± 8.39 (8.82)	60.08 ± 18.77 (14.3)	55.45 ± 12.23 (13.2)
Decanal	Marine, cucumber, floral, fatty, orange, green	14.86 ± 3.32 (7.43)	18.25 ± 4.73 (9.12)	24.51 ± 2.67 (12.25)
2,3-Octanedione	Metallic	76.62 ± 16.84 (6.38)	91.13 ± 16.81 (7.59)	131.99 ± 32.16 (11.00)
1-Heptanol	Green, fermented, nutty	33.90 ± 6.47 (6.28)	36.95 ± 9.34 (6.84)	45.02 ± 5.32 (8.34)
Undecanal	Green, aniseed, fruity, minty	17.19 ± 4.39 (3.44)	22.03 ± 9.36 (4.41)	25.42 ± 5.16 (5.08)
2-Ethyl-Furan	Rubber, pungent, green bean	7.91 ± 1.81 (3.44) ^a^	15.02 ± 1.82 (6.53) ^ab^	16.92 ± 0.30 (7.35) ^b^
(E)-2-Octenal	Moldy, pungent, cucumber, fatty, mushroom	9.76 ± 1.67 (3.25) ^a^	11.52 ± 1.65 (3.84) ^ab^	18.12 ± 1.90 (6.04) ^b^
Pentanal	Acetaldehyde-like, pungent	11.99 ± 3.08 (1.85)		79.12 ± 9.15 (8.79)
3,5-octadien-2-one	Green, floral, cucumber	246.58 ± 34.50 (1.64)	188.64 ± 95.07	243.43 ± 28.08 (1.62)
2-Pentyl-furan	Liquorice, orange	5.02 ± 0.76 ^a^	8.48 ± 1.11 (1.41) ^ab^	12.04 ± 0.94 (2.01) ^b^
(E, E)-2,4-Heptadienal	Fishy, grassy	14.59 ± 0.75	16.00 ± 1.24 (1.11)	14.49 ± 2.66
Total odor-activecompounds (TOAC)		896.13 ± 72.31 ^a^	1434.96 ± 71.22 ^b^	2482.82 ± 197.89 ^c^
∑n-3 derived ^3^		65.33 ± 4.60 ^a^	103.32 ± 10.23 ^b^	102.35 ± 10.18 ^b^
∑n-6 derived ^4^		325.43 ± 119.35 ^a^	723.85 ± 68.21 ^ab^	1228.49 ± 206.39 ^b^
∑n-9 derived ^5^		379.67 ± 85.12 ^a^	572.3 ± 106.38 ^ab^	865.25 ± 82.68 ^b^

Values (mean ± standard error, *n* = 4, N = 16) in the same row with different superscripts are significantly different (*p* < 0.05; Tukey’s test). ^1^ Odor-active compounds are volatile compounds with odor activity values—OAV = C_i_/OT_i_, where C_i_ is the estimated concentration of the volatile compound, and OT_i_ is the odor threshold of the compound reported in the literature. ^2^ Odor description from the study by Liu et al. [18]. ^3^ ∑n-3 derived: sum of 2-hexenal, (Z)-4-heptenal, (E, E)-2,4-heptadienal, (E, Z)-2,6-nonadienal, 2-Ethyl-furan, which are derived from n-3 fatty acids. ^4^ ∑n-6 derived: sum of 1-octen-3-ol, 2-octen-1-ol, pentanal, hexanal, heptanal, (E)-2-heptenal, (E)-2-octenal, (E)-2-nonenal, 2-Pentyl-furan, which are derived from n-6 fatty acids. ^5^ ∑n-9 derived: sum of 1-heptanol, heptanal, octanal, nonanal, which are derived from n-9 fatty acids.

**Table 4:** Fillet tastes of triploid rainbow trout fed the diets with different substitution levels.

Taste-Related Compounds	Taste Description ^1^	F100	F50	F0
		mg/100 g Muscle (TAV ^2^)		
AMP ^3^	Umami	6.88 ± 0.24 (0.14)	6.82 ± 0.64 (0.14)	6.66 ± 0.37 (0.13)
IMP ^3^	Umami	63.77 ± 7.11 (2.55) ^a^	94.03 ± 14.36 (3.76) ^b^	84.01 ± 12.87 (3.36) ^ab^
GMP ^3^	Umami	0.35 ± 0.01 (0.03) ^b^	0.25 ± 0.04 (0.02) ^a^	0.28 ± 0.02 (0.02) ^ab^
Aspartic acid	Umami	0.11 ± 0.01 (0.001) ^c^	0.09 ± 0.01 (0.001) ^b^	0.05 ± 0.00 (0.001) ^a^
Glutamic acid	Umami	19.99 ± 0.66 (0.67) ^a^	28.83 ± 0.76 (0.96) ^c^	24.68 ± 1.03 (0.82) ^b^
Sum of umami taste compounds (SUTC)		112.63 ± 7.44 ^a^	162.52 ± 14.37 ^b^	141.77 ± 14.58 ^ab^
EUC (g/100 g) ^4^		1.73 ± 0.01 ^a^	1.85 ± 0.03 ^b^	1.76 ± 0.03 ^ab^
Threonine	Sweet	1.21 ± 0.05 (0.005)	1.39 ± 0.08 (0.005)	1.19 ± 0.07 (0.005)
Serine	Sweet	3.87 ± 0.31 (0.03) ^a^	6.19 ± 0.50 (0.04) ^b^	3.27 ± 0.18 (0.02) ^a^
Glycine	Sweet	47.47 ± 2.58 (0.36) ^b^	45.51 ± 2.61 (0.35) ^b^	28.78 ± 1.31 (0.22) ^a^
Alanine	Sweet	31.23 ± 0.41 (0.52) ^a^	35.52 ± 0.48 (0.59) ^b^	36.54 ± 0.99 (0.61) ^b^
Proline	Sweet	10.11 ± 1.39 (0.03)	7.44 ± 0.44 (0.02)	8.50 ± 0.88 (0.03)
Lysine	Sweet	1.57 ± 0.08 (0.03)	1.66 ± 0.07 (0.03)	1.74 ± 0.07 (0.03)
Sum of sweet taste compounds (SWTC)		95.00 ± 2.84 ^b^	97.71± 3.24 ^b^	80.03 ± 2.23 ^a^
Valine	Bitter	8.86 ± 0.31 (0.22) ^a^	10.91 ± 0.38 (0.27) ^b^	11.30 ± 0.45 (0.28) ^b^
Methionine	Bitter	5.72 ± 0.20 (0.19)	5.38 ± 0.22 (0.18)	5.46 ± 0.16 (0.18)
Leucine	Bitter	7.45 ± 0.23 (0.04) ^a^	9.87 ± 0.31 (0.05) ^b^	9.59 ± 0.45 (0.05) ^b^
Isoleucine	Bitter	2.45 ± 0.10 (0.03) ^a^	3.38 ± 0.11 (0.04) ^b^	3.59 ± 0.20 (0.04) ^b^
Arginine	Bitter	3.34 ± 0.22 (0.07) ^a^	3.56 ± 0.20 (0.07) ^a^	5.80 ± 0.58 (0.12) ^b^
Histidine	Bitter	51.87 ± 1.06 (2.61) ^a^	57.36 ± 1.27 (2.87) ^b^	48.20 ± 0.86 (2.41) ^a^
Phenylalanine	Bitter	4.16 ± 0.17 (0.05)	4.76 ± 0.27 (0.05)	4.08 ± 0.25 (0.05)
Tyrosine	Bitter	9.08 ± 0.44	10.00 ± 0.39	10.35 ± 0.39
Sum of bitter taste compounds (SBTC)		93.31 ± 1.86 ^a^	105.21 ± 1.18 ^b^	98.36 ± 9.48 ^ab^
Oxalic acid	Sour	401.05 ± 26.78 (7.96)	370.12 ± 6.62 (7.34)	354.14 ± 14.13 (7.03)
Lactic acid	Sour	499.79 ± 24.43 (3.97)	536.77 ± 37.78 (4.26)	514.41 ± 16.16 (4.08)
Maleic acid	Sour	15.17 ± 1.09 ^b^	13.78 ± 0.28 ^ab^	11.80 ± 0.49 ^a^
Succinic acid	Sour	86.48 ± 5.93 (8.65)	119.60 ± 8.79 (11.96)	96.89 ± 22.28 (9.69)
Sum of sour taste compounds (SOTC)		1002.49 ± 48.25	1040.28 ± 34.89	977.24 ± 39.96
Na^+^	Saline	51.66 ± 9.37 (0.29)	33.22 ± 2.19 (0.18)	41.85 ± 3.74 (0.23)
K^+^	Saline	504.70 ± 43.68 (3.88)	461.40 ± 11.72 (3.55)	463.79 ± 16.76 (3.57)
Ca^2+^	Saline	19.49 ± 6.15 (0.13) ^a^	28.86 ± 9.66 (0.19) ^ab^	54.84 ± 7.86 (0.37) ^b^
Mg^2+^	Saline	33.46 ± 0.96 (0.35)	31.79 ± 0.59 (0.33)	32.48 ± 1.31 (0.34)
Cl^−^	Saline	235.50 ± 8.76 (1.81) ^a^	254.93 ± 3.87 (1.96) ^ab^	273.23 ± 1.69 (2.10) ^b^
PO_4_^3−^	Saline	100.08 ± 4.79 (0.77)	113.14 ± 3.30 (0.87)	109.81 ± 1.39 (0.84)
Sum of saline taste compounds (SATC)		944.89 ± 52.23	923.33 ± 11.83	976.00 ± 20.90

Values (mean ± standard error, *n* = 4, N = 16) in the same row with different superscripts are significantly different (*p* < 0.05; Tukey’s test). ^1^ Taste descriptions from the literature: Liu et al. [18]. ^2^ TAV—the taste activity value; TAV = C_i_/TT_i_, where C_i_ is the estimated concentration of the taste-related compound, and TT_i_ is the taste threshold of the compound reported in the literature. ^3^ AMP—adenosine-5′-monophosphate; IMP—inosine-5′-monophosphate; GMP—guanosine-5′-monophosphate. ^4^ EUC—the equivalent umami concentration; EUC (gMSG/100 g) = Σa_i_b_i_ + 1218 (Σa_i_b_i_) (Σa_j_b_j_) [18].

**Table 5:** Fillet nutritional values of triploid rainbow trout fed the diets with different substitution levels.

Fatty Acid Compositions	F100	F50	F0
	mg/g Muscle		
C12:0	0.01 ± 0.00	0.02 ± 0.00	0.01 ± 0.00
C14:0	0.89 ± 0.01 ^c^	0.77 ± 0.05 ^b^	0.44 ± 0.02 ^a^
C16:0	4.62 ± 0.06 ^b^	5.23 ± 0.16 ^c^	3.71 ± 0.17 ^a^
C18:0	1.81 ± 0.02 ^b^	2.11 ± 0.13 ^c^	1.29 ± 0.01 ^a^
C20:0	0.11 ± 0.00 ^b^	0.12 ± 0.01 ^b^	0.08 ± 0.00 ^a^
C22:0	0.07 ± 0.00 ^b^	0.07 ± 0.01 ^b^	0.05 ± 0.00 ^a^
C24:0	0.03 ± 0.00 ^b^	0.02 ± 0.00 ^b^	0.01 ± 0.00 ^a^
C16:1n-7	1.21 ± 0.02 ^b^	1.05 ± 0.06 ^b^	0.50 ± 0.16 ^a^
C18:1n-9(Z)	6.90 ± 0.10 ^a^	10.1 ± 0.38 ^b^	8.00 ± 0.77 ^ab^
C18:1n-9(E)	1.08 ± 0.01 ^b^	1.26 ± 0.08 ^c^	0.75 ± 0.01 ^a^
C20:1n-9	1.16 ± 0.05 ^b^	0.98 ± 0.06 ^b^	0.74 ± 0.02 ^a^
C22:1n-9	0.16 ± 0.00 ^a^	0.19 ± 0.02 ^b^	0.16 ± 0.00 ^a^
C24:1n-9	0.21 ± 0.06	0.07 ± 0.01	0.06 ± 0.01
C18:2n-6	6.05 ± 0.16 ^a^	9.28 ± 0.42 ^b^	5.99 ± 0.36 ^a^
C20:2n-6	0.35 ± 0.01 ^b^	0.46 ± 0.03 ^c^	0.26 ± 0.01 ^a^
C20:3n-6	0.12 ± 0.01 ^a^	0.22 ± 0.02 ^c^	0.16 ± 0.00 ^b^
C20:4n-6	0.13 ± 0.00 ^b^	0.12 ± 0.01 ^b^	0.10 ± 0.00 ^a^
C18:3n-3	0.05 ± 0.00	0.10 ± 0.01	0.10 ± 0.02
C20:5n-3 (EPA)	0.72 ± 0.06 ^b^	0.59 ± 0.05 ^b^	0.38 ± 0.01 ^a^
C22:6n-3 (DHA)	2.26 ± 0.19 ^b^	1.77 ± 0.07 ^ab^	1.44 ± 0.06 ^a^
EPA + DHA	2.98 ± 0.25 ^b^	2.36 ± 0.12 ^ab^	1.82 ± 0.07 ^a^
SFA^1^	7.54 ± 0.07 ^b^	8.34 ± 0.35 ^c^	5.59 ± 0.16 ^a^
MUFA^1^	10.72 ± 0.13 ^a^	13.64 ± 0.61 ^b^	10.22 ± 0.63 ^a^
PUFA^1^	9.67 ± 0.42 ^a^	12.53 ± 0.60 ^b^	8.59 ± 0.43 ^a^
PUFA/SFA	1.28 ± 0.05	1.50 ± 0.01	1.53 ± 0.06
LC-PUFA ^1^	3.23 ± 0.25 ^b^	2.70 ± 0.14 ^ab^	2.09 ± 0.08 ^a^
∑n-3 ^1^	3.03 ± 0.25 ^b^	2.45 ± 0.13 ^ab^	1.91 ± 0.08 ^a^
∑n-6 ^1^	6.64 ± 0.18 ^a^	10.08 ± 0.48 ^b^	6.52 ± 0.37 ^a^
∑n-3/∑n-6	0.45 ± 0.03 ^b^	0.24 ± 0.00 ^a^	0.30 ± 0.01 ^a^
AI ^2^	0.49 ± 0.01 ^b^	0.40 ± 0.00 ^a^	0.36 ± 0.02 ^a^
TI ^3^	0.40 ± 0.02	0.42 ± 0.00	0.37 ± 0.01

Values (mean ± standard error, *n* = 4, N = 16) in the same row with different superscripts are significantly different (*p* < 0.05; Tukey’s test). ^1^ SFA—saturated fatty acids; MUFA—monounsaturated fatty acids; PUFA—polyunsaturated fatty acids; LC-PUFA—long chain polyunsaturated fatty acids; n-3—n-3 polyunsaturated fatty acids; n-6—n-6 polyunsaturated fatty acids. ^2^ AI: atherogenic index = (C12:0 + C14:0 + C16:0)/(Sum PUFAs + Sum MUFAs). ^3^ TI—thrombogenic index = (C14:0 + C16:0 + C18:0)/(0.5 × Sum PUFAs + 0.5 × Sum n-6 PUFAs + 3 × Sum n-3 PUFAs + n-3 PUFAs/n-6 PUFAs).

## Data Availability

The original contributions presented in the study are included in the article, further inquiries can be directed to the corresponding author.

## Appendix A

**Table A1:** Formulation and proximate composition of the experimental diets (% dry matter).

Ingredients	F100	F50	F0
Fish meal ^1^	60.0	30.0	0.0
Soybean meal ^1^	0.0	11.0	22.0
Soy protein concentrate ^1^	0.0	5.0	10.0
Corn gluten meal ^1^	0.0	4.5	9.0
Wheat gluten ^1^	0.0	4.0	8.0
Blood cell powder ^1^	0.0	5.0	10.0
Wheat meal ^1^	13.5	13.5	13.5
Cellulose	5.12	2.57	0.0
Fish oil	18.6	10.25	0.00
Canola oil	0.00	10.25	22.52
Mineral and vitamin premix ^2^	1.00	1.00	1.00
Ca(H_2_PO_4_)_2_	0.80	0.80	0.80
Choline chloride	0.30	0.30	0.30
Mold inhibitor	0.10	0.10	0.10
Ethoxyquin	0.05	0.05	0.05
betaine	0.50	0.50	0.50
Astaxanthin ^3^	0.03	0.03	0.03
Lysine HCL (75%)	0.00	0.80	1.50
DL-methionine	0.00	0.35	0.70
Total	100	100	100
Proximate analysis			
Crude protein (% dry matter)	45.6	45.5	45.3
Crude lipid (% dry matter)	23.0	23.3	23.2
Lysine (% dry matter)	2.97	3.04	2.98
Methionine (% dry matter)	1.00	1.01	1.01
Threonine (% dry matter)	1.47	1.50	1.53
Arginine (% dry matter)	2.13	2.09	2.04
Fatty acids (% of identified fatty acids)			
C14:0	3.67	2.12	1.71
C16:0	16.8	13.4	12.0
C18:0	4.08	3.61	3.27
C16:1n-7	3.42	2.10	1.63
C18:1n-9	21.2	28.2	31.5
C20:1n-9	2.71	2.24	2.70
C22:1n-9	3.53	1.57	2.27
C18:2n-6	25.7	33.5	33.0
C20:4n-6	1.38	0.84	0.84
C18:3n-3	4.02	5.47	5.99
C20:5n-3	5.37	3.34	2.22
C22:6n-3	8.07	3.87	3.04
Formula cost saving rate (%, compared with F100 diet)	-	24.6%	50.7%

^1^ Fish meal, crude protein 72%, crude lipid 8.7%; soybean meal, crude protein 53.5%, crude lipid 3.1%; soy protein concentrate, crude protein 70.2%, crude lipid 0.5%; corn gluten meal, crude protein 67.7%, crude lipid 3.1%; wheat gluten, crude protein 84.6%, crude lipid 2.0%; blood cell powder, crude protein 95.2%, crude lipid 1.7%; wheat meal, crude protein 17.6%, crude lipid 1.5%. ^2^ Both of these ingredients were supplied by Tongwei Co., Ltd., China. Vitamin premix, vitamin premix for carnivorous fish: 0.5%; Mineral premix, mineral premix for carnivorous fish: 0.5%. ^3^ Astaxanthin, the effective content is 10% (CAROPHYLL^®^, DSM, Heerlen, The Netherlands).
